# CD44 Splice Variant v8-10 as a Marker of Serous Ovarian Cancer Prognosis

**DOI:** 10.1371/journal.pone.0156595

**Published:** 2016-06-02

**Authors:** Amanda Sosulski, Heiko Horn, Lihua Zhang, Caroline Coletti, Vinod Vathipadiekal, Cesar M. Castro, Michael J. Birrer, Osamu Nagano, Hideyuki Saya, Kasper Lage, Patricia K. Donahoe, David Pépin

**Affiliations:** 1 Pediatric Surgical Research Laboratories, Massachusetts General Hospital, Department of Surgery, Boston, Massachusetts, United States of America; 2 Department of Surgery, Harvard Medical School, Boston, Massachusetts, United States of America; 3 Broad Institute, Cambridge, Massachusetts, United States of America; 4 Gynecological Oncology, Massachusetts General Hospital, Department of Medicine, Boston, Massachusetts, United States of America; 5 Harvard Medical School, Boston, Massachusetts, United States of America; 6 Division of Gene Regulation, Institute for Advanced Medical Research, School of Medicine, Keio University, 35 Shinanomachi, Shinjuku-ku, Tokyo, Japan; 7 Core Research for Evolutional Science and Technology (CREST), Japan Science and Technology Agency, Tokyo, Japan; National Cancer Center, JAPAN

## Abstract

CD44 is a transmembrane hyaluronic acid receptor gene that encodes over 100 different tissue-specific protein isoforms. The most ubiquitous, CD44 standard, has been used as a cancer stem cell marker in ovarian and other cancers. Expression of the epithelial CD44 variant containing exons v8-10 (CD44v8-10) has been associated with more chemoresistant and metastatic tumors in gastrointestinal and breast cancers, but its role in ovarian cancer is unknown; we therefore investigated its use as a prognostic marker in this disease. The gene expression profiles of 254 tumor samples from The Cancer Genome Atlas RNAseqV2 were analyzed for the presence of CD44 isoforms. A trend for longer survival was observed in patients with high expression of CD44 isoforms that include exons v8-10. Immunohistochemical (IHC) analysis of tumors for presence of CD44v8-10 was performed on an independent cohort of 210 patients with high-grade serous ovarian cancer using a tumor tissue microarray. Patient stratification based on software analysis of staining revealed a statistically significant increase in survival in patients with the highest levels of transmembrane protein expression (top 10 or 20%) compared to those with the lowest expression (bottom 10 and 20%) (p = 0.0181, p = 0.0262 respectively). Expression of CD44v8-10 in primary ovarian cancer cell lines was correlated with a predominantly epithelial phenotype characterized by high expression of epithelial markers and low expression of mesenchymal markers by qPCR, Western blot, and IHC. Conversely, detection of proteolytically cleaved and soluble extracellular domain of CD44v8-10 in patient ascites samples was correlated with significantly worse prognosis (p<0.05). Therefore, presence of transmembrane CD44v8-10 on the surface of primary tumor cells may be a marker of a highly epithelial tumor with better prognosis while enzymatic cleavage of CD44v8-10, as detected by presence of the soluble extracellular domain in ascites fluid, may be indicative of a more metastatic disease and worse prognosis.

## Introduction

Ovarian cancer is the most common cause of death among women with gynecological cancer worldwide, with the high mortality attributable to the advanced stage at which it is diagnosed, typically long after intraperitoneal metastatic spread has occurred [1]. In order for metastasis to occur, ovarian cancer cells often alter expression of proteins involved in extracellular matrix interaction, such as CD44, to modulate invasion [2, 3]. As a cellular adhesion molecule and a major extracellular glycoprotein, the hyaluronic acid receptor CD44 has been implicated in tumor invasion and metastasis in breast, lung, and gastrointestinal cancers [2].

CD44 is encoded by a maximum of 20 exons, 10 of which are referred to as variant exons. The standard isoform (CD44s) contains only 10 exons, including the first 5 exons of the 5’ end and the last 5 exons of the 3’ end, thus lacking the variant exons in the middle, referred to as v1-v10 or exons 5a-15 in standard nomenclature [1, 4, 5]. CD44s is the most ubiquitous isoform, widely expressed on the surface of most tissues and all hematopoieitic cells where it is responsible for lymphocyte homing and other cell-cell and cell-extracellular matrix interactions. A large number of alternative splicing isoforms of CD44 exist which contain a combination of variant exons (V1 through V10) inserted into the juxtamembrane extracellular region under the control of epithelial splicing regulatory proteins (ESRP) 1 and 2 [6]. Many CD44 variant isoforms have tissue-specific expression; CD44v8-10 is expressed in epithelial tissues and in epithelial-type carcinomas of the pancreas, prostate, breast, and lung [7], where it has been extensively studied as a specific marker of malignant cells, with increased expression observed during gastric carcinogenesis in a murine model and in the adenoma to carcinoma progression in human colorectal cancer [8–10] and gastric cancers [11].

CD44s and CD44 variant isoforms have been implicated in drug resistance and identified as stem cell markers [12]. In ovarian cancer, studies using expression of CD44s and its variant isoforms in evaluating prognosis have produced contradictory results, with some showing decreased prognosis, and others improved prognosis, or no effect [13]. However, studies on isoforms containing different individual variant exons in ovarian cancer have generally suggested improved overall survival associated with splice isoforms of CD44 [14]. The epithelial splice variant 8–10, expressing v8, v9, and v10 together, has not been examined in high-grade serous ovarian cancer; Therefore, it’s reliability and feasibility in predicting prognosis was investigated in this study.

## Materials and Methods

### The Cancer Genome Atlas (TCGA) data analysis

Expression data (Level 3) on documented CD44s and CD44 variant isoforms for tumors of the ovarian serous cystadenocarcinoma was analyzed in a cohort of 255 subjects in the TCGA (after censoring samples from recurrent tumors and focusing only on high-grade disease (G2 and G3)). We used Level 3 data to avoid re-analyzing the raw datasets; for further information please see the original publication [15]. Based on the annotation file for the RNAseqV2 analysis (S1 Table), we defined a CD44 isoform as v8-10 containing the exons chr11:35229652–35229753:+, chr11:35231512–35231601:+ and chr11:35232793–35232996:+. Survival analysis of the top and bottom 10% based on the mean expression of the three (v8-10) exons, was run using the “survival” and “survMisc” package in R. Specifically, we used the supremum (Renyi) version of the log-rank test which is designed for the analysis of crossing survival curves.

### Tumor Tissue Micro-array Analyses

A second cohort of 210 high-grade ovarian serous cystadenocarcinoma patients hospitalized at the Massachusetts General Hospital and Brigham and Women's Hospital Dana Farber Cancer Center between 1993–2009 [16] were studied under internal review board (IRB) approved protocols (MGH2007P00001918 or DFCI 02–051). Tumor core samples (3 mm) were obtained at initial debulking surgery, scored according to the Fédération Internationale de Gynécologie Obstétrique (FIGO) guidelines, and were formalin-fixed and paraffin-embedded. All tumors biopsies, discarded de-identified ascites samples, and corresponding patient information were collected under IRB-approved protocols after written informed consent was obtained. Each new paraffin block contained 24 randomly distributed tumor cores, with at least 2 cores per patient, and were sectioned at 7 micron thickness as previously described [17]. Immunohistochemistry was performed on slides with tumor microarray (TMA) sections as previously published [16] before being blocked using a solution of 1% bovine serum albumin (BSA) with 2% donkey serum in phosphate buffered saline (PBS) for 1 hour at room temperature, incubated with an antibody to CD44v8-10 (CD44v9 RV3) [4] at 1:12,500 overnight in a humidified chamber at 4C, washed with PBS, and incubated using a secondary goat anti-rat horseradish peroxidase (HRP) conjugated antibody (Santa Cruz biotechnology, Dallas TX) at 1:200 for 1.5 hours at room temperature. Slides were rinsed and stained with a diaminobenzidine (DAB) solution, counter stained with Hematoxylin (Dako, Carpinteria CA), dehydrated, and coverslipped before being scanned and digitized with a Scanscope (Leica, Buffalo Grove IL) and analyzed with the Aperio Imagescope software (Leica, Buffalo Grove IL). Positivity and Intensity (PI) scores were calculated as the product of stained area fraction (positivity) and its intensity. For the purpose of the staining analysis, we examined only cores in which greater than 50% of the tissue remained intact on the glass slide. Based on this criterion we had 6 patients for whom we calculated the PI score using a single core, and 203 in which we took the average PI score from 2 cores, for a total of 209 patients analysed. Patient survival was calculated from date of diagnosis to date of death, or entrance into hospice care when not available and converted from days to months by dividing the number of days by 30. Overall survival was analyzed by Kaplan-Meier plots and statistical significance inferred by log-rank tests. Correlations to clinical parameters were made using chi-square analysis. Statistical analyses were performed using the Prism 6 software (Graphpad, La Jolla CA).

### Cell Lines

A series of primary ovarian cancer cell lines was derived from patient ascites, per IRB-approved protocol (#2007P001918/MGH) [16]. Briefly, after centrifugation of the ascites fluid, the cell pellets were introduced into tissue culture under two different conditions (adherent and spheroid). In adherent cultures, cells were maintained at complete confluency for several weeks to several months until tight epithelial cell clones were observed. Contaminating cell types (leukocytes, mesothelial, and fibroblast cells) were removed by repeated partial trypsin digestion until no contaminating cell types could be observed. Subsequently the cells were passaged at least 5 times at 1:10 dilution to derive stable homogenous cell line. Alternatively, for spheroid cultures (denoted by the–sph suffix), cells were plated in regular flasks for 24h and non-adherent cells were collected and transferred into new ultra-low attachment flasks, while attached cells were discarded. Spheroid cultures were passaged by dissociation and diluted 1:10 for at least 5 passages to derive stable homogenous cell lines. The panel of primary cell lines consists of 16 serous epithelial ovarian cancers (ptAB, ptAB-sph, ptAF, ptAF-sph, ptW, ptW-sph, ptAO, ptAO-sph, ptD, ptH, ptAI, PKD1, PKD2, ptAL-sph, ptAM-sph, ptAK-sph), one endometrioid epithelial cancers (ptAP-sph), and one mucinous epithelial ovarian cancer (ptG) grown in adherent and/or spheroid culture conditions.

Primary cell lines were maintained in cell culture in monolayers in DMEM:F12 media with 10% FFBS 1% penicillin/streptomycin, 1% L-glutamine (Corning, New York NY) or as non-adherent spheres in serum-free tumor sphere media [16] at 37C in a humidified atmosphere containing 5% CO2. Additionally, ovarian cancer established cell lines OVCAR5 [18] and OVCAR8 [19], were maintained in DMEM media with 10% fetal bovine serum (FBS) 1% penicillin/streptomycin, 1% L-glutamine (Corning, New York NY).

### Q-PCR and Principle Component Analysis

Quantitative PCR was performed on primary cell lines derived from patients’ ascites (ptAB, ptAB-sph, ptAF, ptAF-sph, ptW, ptW-sph, ptAO-sph, ptG, ptD, ptH, PKD1, PKD2, ptAL-sph, ptAP-sph, ptAM-sph) and on the established epithelial ovarian cancer cell lines OVCAR-5 and OVCAR-8. Cell pellets were obtained after centrifugation, washed with phosphate buffered saline (PBS), and frozen at -80C, until RNA extraction using a Qiagen RNeasy Mini Kit (Germantown, MD) and quantitated by Nanodrop. cDNA was reverse transcribed using 500ng of RNA per sample with Superscript III First Strand Synthesis Super Mix (Invitrogen, Carlsbad CA), and qPCR was performed for established makers of epithelial to mesenchymal transformation (EMT), and pluripotency including CD44s, CD44v8-10 [20], vimentin, E-cadherin, ESRP1, Zeb1, Lin-28, Sox-2, Oct-4, N-cadherin, and EpCAM, with GAPDH as an internal standard. Relative expression was inferred by the determination of cycle threshold (Ct) and calculated using 2^-ΔCt transformation normalized to GAPDH Ct. Principle component analysis (PCA) was performed using these gene expression values after log transformation using the “FactoMineR” package in R. PCA with mesenchymal as well as epithelial markers clearly separated on the first component, while EMT and pluripotent markers were perpendicular on the second component, but indistinguishable from each other.

### Western Analysis

Cell pellets harvested from a panel of primary patient ascites cell lines (ptAB, ptAB-sph, ptAF, ptAF-sph, ptW, ptW-sph, ptAO, ptAO-sph, ptD, ptH, ptAI, PKD1, PKD2, ptAL-sph, ptAP-sph, ptAM-sph, ptAK-sph) and 2 established cancer cell lines (OVCAR-5 and OVCAR-8) were washed twice with 10ml ice cold PBS and whole cell extracts prepared in 1x lysis buffer (Cell Signaling Technology, Danvers MA) in sterile water containing a protease inhibitor cocktail (Roche Indianapolis, IN) and phophatase inhibitor tablets (Roche, Indianapolis IN). Total protein per lysate was obtained using a Pierce bicinchoninic acid assay (BCA) protein quantification kit (Thermoscientific, Rockford IL), and equal amounts of proteins (15ug) reduced, loaded in each lane, and separated by NuPage 4–12% gels (Life technologies, Carlsbad CA). Proteins were transferred to Immobilon polyvinylidene difluoride (PVDF) membranes (Millipore, Billerica MA), blocked in 5% non-fat milk dissolved in a tris-buffered saline and tween 20 (TBST) solution, and then incubated overnight at 4C to detect full length transmembrane or intracellular protein with primary antibody rat anti CD44v8-10 (RV3) (1:1000) [8], or mouse anti CD44s (R+D, Minneapolis MN) (1:500), rabbit anti Vimentin (ABCAM, Cambridge MA) (1:1000), or rabbit anti- E-cadherin (ABCAM, Cambridge MA)(1:10,000) antibodies, each diluted in 5% nonfat milk in TBST. PVDF membranes were then washed with TBST and incubated with an appropriate secondary antibody, either an anti rabbit (Cell Signaling technology, Danvers MA), anti mouse (Jackson Laboratories, Bar Harbor MA), or anti rat (Santa Cruz biotechnology, Dallas TX) antibody conjugated to a horse radish peroxidase indicator, each at 1:10,000 in 5% milk in TBST solution for 1 hr at room temperature. Membranes were again washed in TBST and treated with Pico ECL™ chemiluminescence substrate for protein visualization (Thermoscientific, Rockford IL). Relative protein abundance was measured by densitometry of the Western blot bands and normalized for equal protein loading using B-actin density by Image J software analysis.

Patient ascites were obtained from fresh therapeutic paracenteses, per an IRB-approved protocol (#2007P001918/MGH). 1 ml of ascites fluid supernatant samples were sonicated, centrifuged at maximum speed for 10 min to clear, and diluted 1:50 in lysis buffer prior to running on a western blot as described above. Membranes were incubated with rat anti CD44v8-10 (RV3) (1:1000) or mouse anti CD44s (R+D, Minneapolis MN) (1:500) overnight at 4C and developed as above using femto ECL™ chemiluminescence substrate (Thermoscientific, Rockford IL) to determine the presence of soluble cleaved and secreted extracellular domain of CD44v8-10 and CD44s shed into patient ascites fluid. As a loading control we used anti-human IgG heavy and light chain horse-radish peroxidase conjugate 1:20,000 (Life Technologies, Carlsbad CA). Images were captured by BioRad XR Gel Doc image lab software (BioRad, Hercules CA) and analyzed by Image-J as a percent of IgG light chain abundance. Shed protein level was then correlated with patient survival, with endpoints being date of death, or entrance into hospice care when not available.

### Immunohistochemistry of epithelial and mesenchymal markers in PDXa explanted tumors

Patient-derived xenografts from ascites (PDXa) where established by implanting 5 million cells of low passage primary lines (ptAP-sph, ptW, ptAM-sph, ptH, ptAB-sph, ptD) resuspended 1:1 in Matrigel (Corning, New York NY) and implanted subcutaneously into the flanks of NOD/SCID/IL2RGamma Jax™ mice (Jackson Laboratory, Bar Harbor ME) under an IACUC-approved experimental protocol (#2009N000033) [16]. OVCAR5 tumors were derived using an identical xenografting protocol using 1 million cells. Resulting tumors were fixed in 4% paraformaldehyde, embedded in paraffin, and sectioned at 7 micrometers thickness onto Fisher Scientific *Probe On plus* microscope slides (Fisher Scientific, New York NY). Slides were deparaffinized in Xylene, rehydrated in alcohol, and microwaved in buffered sodium citrate (0.01M) for antigen retrieval for 5 minutes at high power, 10 minutes at 10% power, and 10 minutes at 20% power, then rinsed in distilled water. Slides were treated with 3% hydrogen peroxide, washed, and blocked using a solution of 1% BSA with 2% fetal calf serum (FCS) in PBS for 1 hour at room temperature, then incubated with CD44v8-10 antibody (RV3 rat monoclonal) (1:12,500), and compared to those incubated with CD44s (R+D, Minneapolis MN, mouse monoclonal 16ug/ml), anti E-cadherin antibody (ABCAM, Cambridge MA, rabbit) (1:500), or anti vimentin antibody (ABCAM, Cambridge MA, rabbit) (1:500) overnight in a humidified chamber at 4C. After incubating with a goat anti-rat HRP antibody (Santa Cruz Biotechnology, Dallas TX), donkey anti-mouse HRP, or donkey anti-rabbit HRP antibody (1:200) (Jackson Laboratories, Bar Harbor MA) for 1.5 hours at room temperature, slides were rinsed and stained with a DAB solution (Sigma Aldrich, St Louis MO), and counter stained with Hematoxylin (Dako, Carpinteria CA), dehydrated, and coverslipped. Representative micrographs were obtained for spatial comparison of proteins previously detected by western analysis of the primary cell lines from which the tumors were grown.

## Results

### Expression of CD44 variant isoforms containing exon 8 to 10 (V8-10) mRNA in The Cancer Genome Atlas (TCGA) RNAseqV2 ovarian cancer dataset

**Fig 1:**
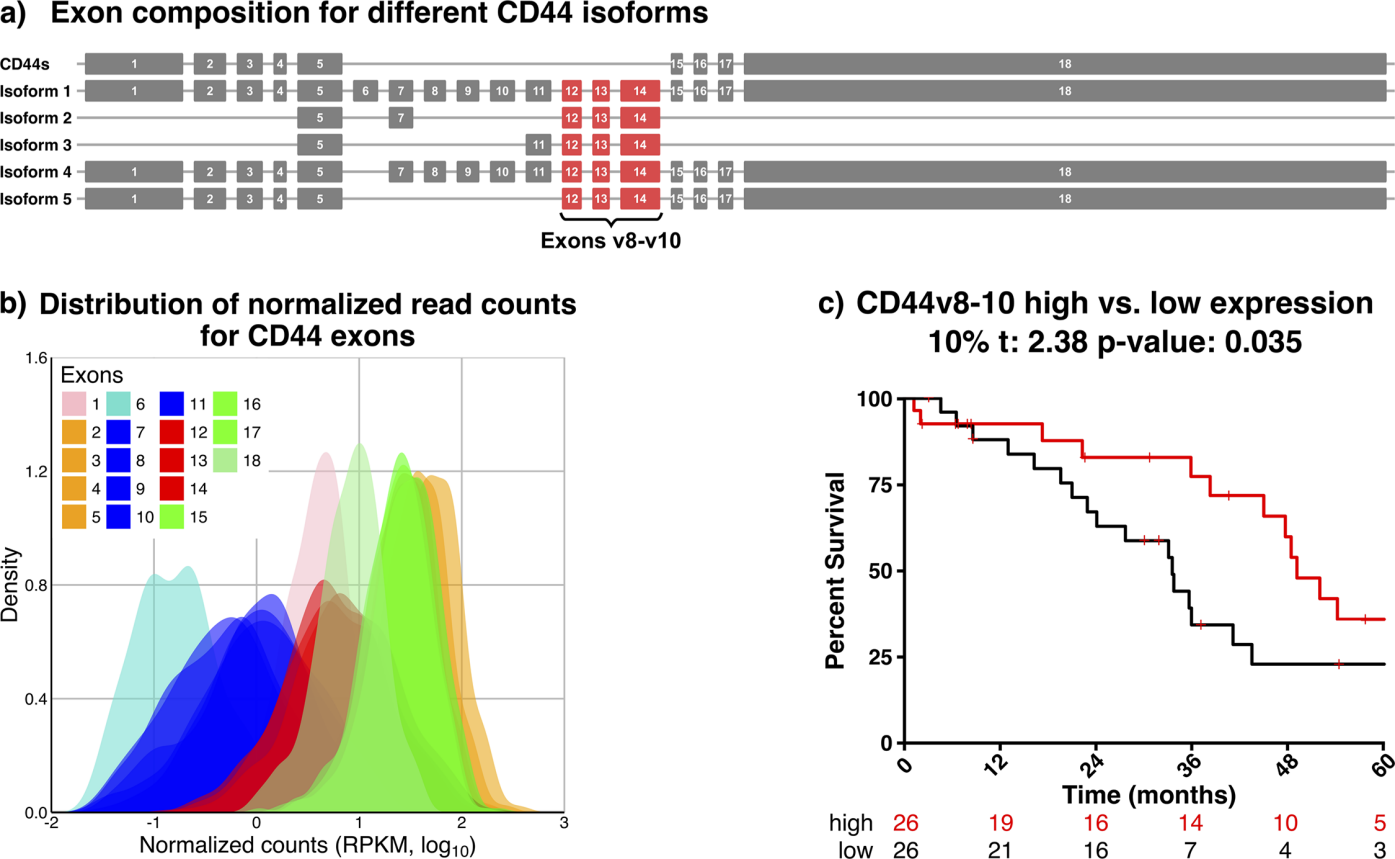
Expression of and survival analysis of CD44 variant 8–10 containing isoforms in the Cancer Genome Atlas Ovarian Serous Cystadenocarcinoma Database. A) Schematic representation of alternative splice isoforms expressing variable exons v8-v10. B) Distribution of RNAseq2 expression data over 254 samples for all CD44 exons in the TCGA ovarian cancer dataset. Samples with no expression were omitted from this figure. C) Kaplan-Meier plot of the survival curve comparing high and low CD44v8-10 expressing samples and corresponding table with the number of patients at each given time point. We defined the high expressing set (red) and the low expressing set (black) as the samples with the highest and lowest expression respectively using the top/bottom 10% of expressers, with the plot extending to 5 years. Bars represent censored data points.

To determine the mRNA expression levels of CD44s and its variant V8-10 containing isoforms (Fig 1A) in serous ovarian tumors, we analyzed the RNAseqV2 resource from TCGA, which includes a cohort of 255 Grade 2 and 3 tumor samples. The expression of individual exons V8, V9, and V10 correlates strongly over all samples, which can also be observed for the “standard” exons 1–5 and 15–18 (S1 Fig), suggesting they are predominantly expressed as a block of v8-10. Furthermore, exons V8-10 appear to be the most abundant of all the variable exons, suggesting cd44v8-10 containing isoforms are the predominant alternatively spliced transcripts of CD44 (Fig 1B). Patients were stratified based on mean mRNA expression levels of exons V8-10 in their tumor and divided into high and low expressing groups (top and bottom 10%), from which and Kaplan-Meier survival curves were generated indicating a significantly better prognosis in high expressers (p = 0.035) (Fig 1C). To determine the relative contribution of each distinct isoform containing exons v8-10 we compared their overall expression and estimated the influence on survival. A trend towards increased survival was observed for all but one CD44 isoforms containing V8-10, although none were significant in isolation (S2 Fig). Multivariate analysis using Cox proportional hazard ratio model found that only age had a significant impact on overall survival (Table 1).

**Table 1:** Multivariate analysis using Cox proportional hazard ratio model in the 255 serous ovarian cancer cohort from The Cancer Genome Atlas examined for CD44v9 expression by RNAseq.

Variable	Hazard ratio	Lower 95% CI	Upper 95% CI	P value	significance
Age	1.0291	1.0109	1.047	0.00151	**
CD44v8-10	0.9129	0.7546	1.087	0.33731	
FIGO stageIIIB	0.9664	0.2727	3.438	0.95788	
FIGO stageIIIc	1.3444	0.4851	3.722	0.56907	
FIGO stageIV	2.1048	0.6727	6.499	0.19806	
Grade 3	1.1439	0.6123	2.121	0.67111	

CD44v8-10 = Standard deviations from the mean of log2 transformed expression level of CD44v8-10 by RNAseq; CI = confidence interval. Significance *<0.05, **<0.01, ***<0.001.

### CD44v8-10 staining in primary tumors is associated with longer survival in an independent cohort of 210 high-grade serous ovarian cancer patients analysed by tumor microarray immunohistochemistry

**Fig 2:**
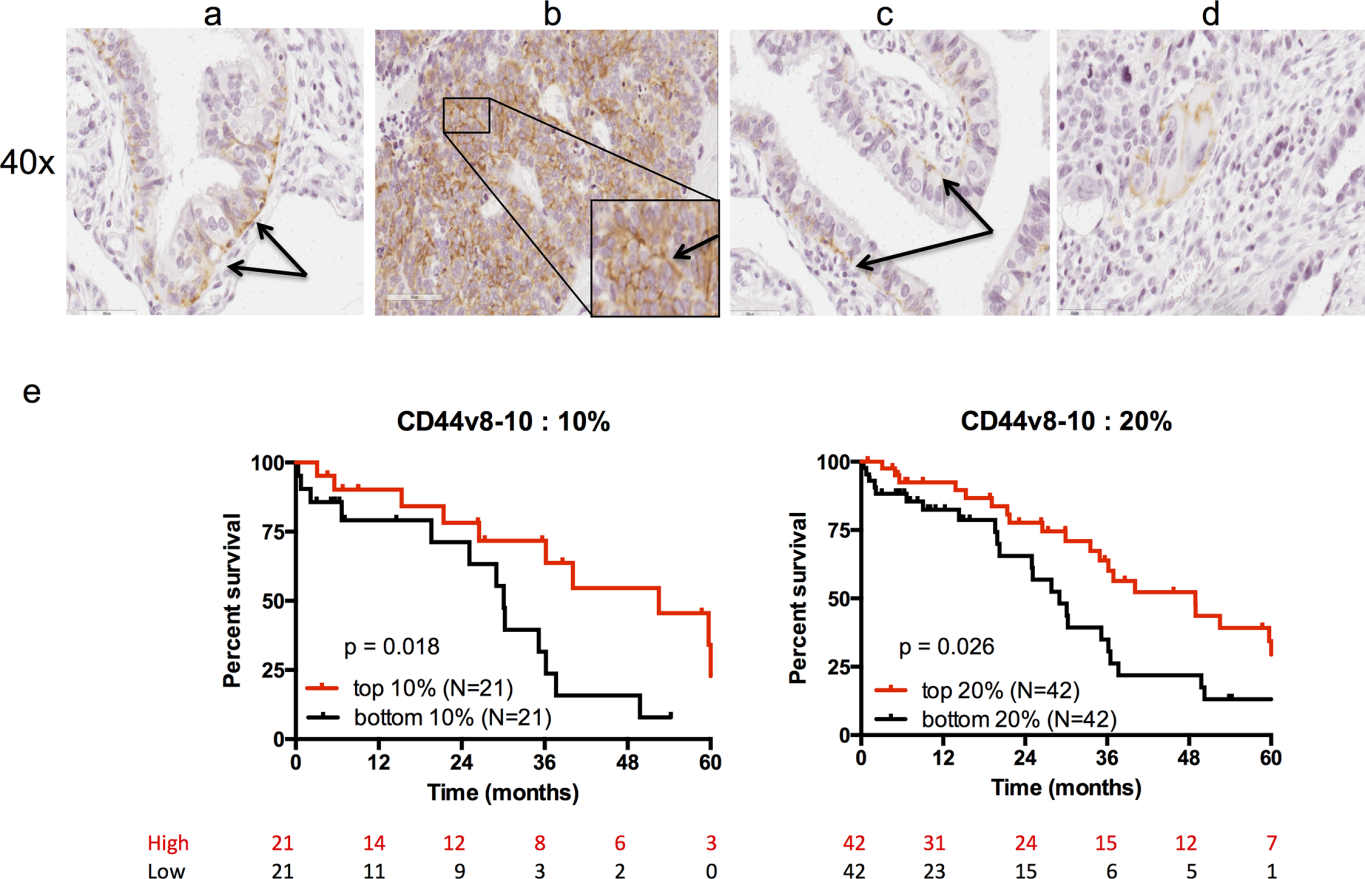
Immunohistochemical and survival analysis of CD44v8-10 expression in High Grade Serous Ovarian Cancer Tumor Tissue Microarrays (TMAs). Fixed paraffin embedded tissue microarray cores were stained by immunohistochemistry using an antibody against CD44v9, scanned using an Aperio Scanscope, and analyzed using Aperio Imagescope software positive pixel count algorithm. Representative images taken at high (40x) power show basal epithelial staining in a and c, more diffuse surface epithelial staining in b, and negative stroma with isolated positive epithelial cells in d. e.) Overall Survival of patients with high-grade serous ovarian carcinoma by CD44 variant expression. Left- Highest and Lowest 10% expression variant and Right- Highest and Lowest 20% of expression of variant, demonstrates significant overall survival in the highest expressers with p = 0.0181 and p = 0.0262, respectively.

To confirm the trends observed in the analysis of the TCGA data suggesting a better prognosis was associated with CD44v8-10 expression, we decided to examine the protein levels of CD44v8-10 in tumor biopsies. Surface expression of the transmembrane CD44v8-10 protein was analyzed in high-grade serous ovarian carcinoma by immunohistochemistry using a specific CD44v8-10 antibody in a tumor microarray containing primary tumor samples from a cohort of 210 patients. All tumors were of high-grade serous histology with the vast majority (71%) scored as at least FIGO Stage III, and Grade 3 (89%) at diagnosis (Table 2). All primary tumor microarray samples examined were taken at the time of initial debulking surgery. Diffuse and focal staining was observed in the columnar epithelium of papillae, often in the basal layer (arrows) (Fig 2A, 2B and 2C) of the epithelium, but not in the tumor stroma in any of the cores (Fig 2D), and always on the cell surface rather than an intracellular or cytoplasmic location (Fig 2B, insert). Total intensity of positive pixels and total number of positive pixels, as determined by Imagescope software, were combined into a positive intensity score (PI), which was averaged across the two representative cores per patient, and used for patient stratification. We chose the top and bottom 10 and 20 percent of patients to compare survival differences between low and high expressers of CD44v8-10 transmembrane surface protein, and found a significant increase in survival for both top 10% and 20% of patients expressing high levels of CD44v8-10, with a median survival of 30 and 29 months when compared to patients expressing the lowest 10% (p = 0.0181) and 20% (p = 0.0262), with a median survival of 49 and 52 months respectively (Fig 2E). The composition of the top and bottom 10% and 20% of patients was similar when examining clinical parameters of FIGO grade, stage, and age (S2 Table), with the exception of a difference in the small number of grade 2 patients. When this population of grade 2 was excluded from the analysis to rule out the influence of these rare lower grade tumors, the survival difference remained significant between the highest and lowest expressers of CD44v8-10 protein (S3 Fig).

**Table 2:** Patient demographics in a high grade serous ovarian cancer tumor tissue microarrays of 210 patients (TMAs).

Characteristic	Total (N = 210)
**Age at diagnosis (years)**	
Mean	62
Median	61.5
Range	(32.6–91.3)
**FIGO Stage, n (%)**	
II	2 (1%)
IIIA or IIIB	44 (21%)
IIIc	105 (50%)
IV	43 (20%)
unknown	16 (8%)
**Grade, n (%)**	
1	0
2	23 (11%)
3	187 (89%)
4	0
**Debulking status, n (%)**	
no residual	86 (41%)
Regional Recurrence	64 (30%)
Never Disease Free	35 (17%)
unknown	25 (12%)

Multivariate analysis of recorded clinical parameters including age, grade, stage, debulking status, and CD44v8-10 staining intensity were analyzed for their effect on prognosis (Table 3).

**Table 3:** Multivariate analysis using a Cox proportional hazard ratio model in the 210 patient cohort analyzed for CD44v8-10 expression by Positive Intensity (PI) score.

Variable	Hazard ratio	Lower 95% CI	Upper 95% CI	p-value	significance
Age	1.0298	1.0132	1.0467	0.00039	***
CD44v8-10	0.7492	0.5926	0.9473	0.01583	*
FIGO stageIIIc	0.7040	0.4428	1.1194	0.13798	
FIGO stageIV	1.4259	0.8031	2.5321	0.22577	
Grade 3	0.8181	0.4583	1.4608	0.49744	
Debulking suboptimal	1.288076	0.768	2.1603	0.33731	
Debulking unknown	0.852278	0.4379	1.6587	0.68027	

CD44v8-10 = Standard deviations from the mean of PI score expression level of CD44v8-10 by IHC; FIGO = stage; CI = confidence interval. Significance *<0.05, **<0.01, ***<0.001.

**Fig 3:**
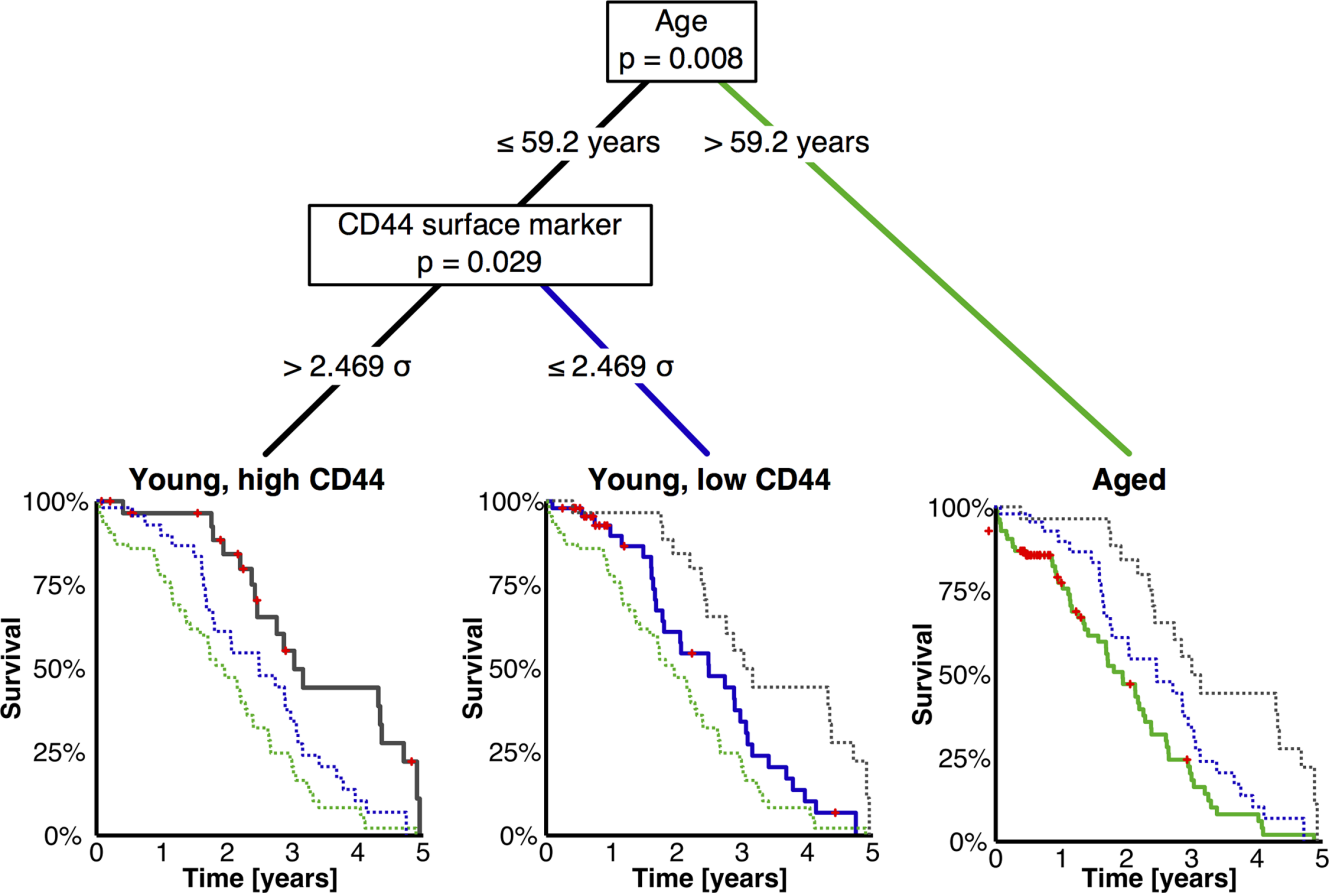
Binary decision tree of clinical variables and CD44v9 staining and their effect on prognosis. The patients from the tumor array sampleset were stratified using Age, CD44v9 staining intensity (σ of CD44v9 expression), grade, debulking status, and FIGO score. Multivariate analysis was performed and effect sized ranked to generate a binary decision tree to maximize prognostic power in an unbiased manner and assign statistical significance. Only statistically significant variables (age and CD44v9 staining) are shown along with their effect on overall patient survival on Kaplan-Meier plots. Bars represent censored data points.

Age had the strongest influence on prognosis followed by CD44v9 staining intensity of the tumor while FIGO staging or grading and debulking status were not significant. Since the cox proportional hazard ratio (Table 3) showed a dependency for age and CD44v8-10 surface marker expression, we wanted to explore variable correlation using an unbiased patient stratification to generate a decision tree which maximises prognostic differences (Fig 3). We stratified patient groups based on age, FIGO stage, Grading, debulking status, and CD44v8-10 expression. The resulting classification showed that patients of >59.2 years of age do not significantly benefit from higher CD44 expression. Younger patients show a strong correlation of better survival when having high CD44 surface marker. These observations refine the findings from the cox hazard ratio model and define a decision tree that could be used clinically to maximise the prognostic power of CD44v8-10 tumor staining (Fig 3).

### Expression of CD44v8-10 correlates with an epithelial phenotype

**Fig 4:**
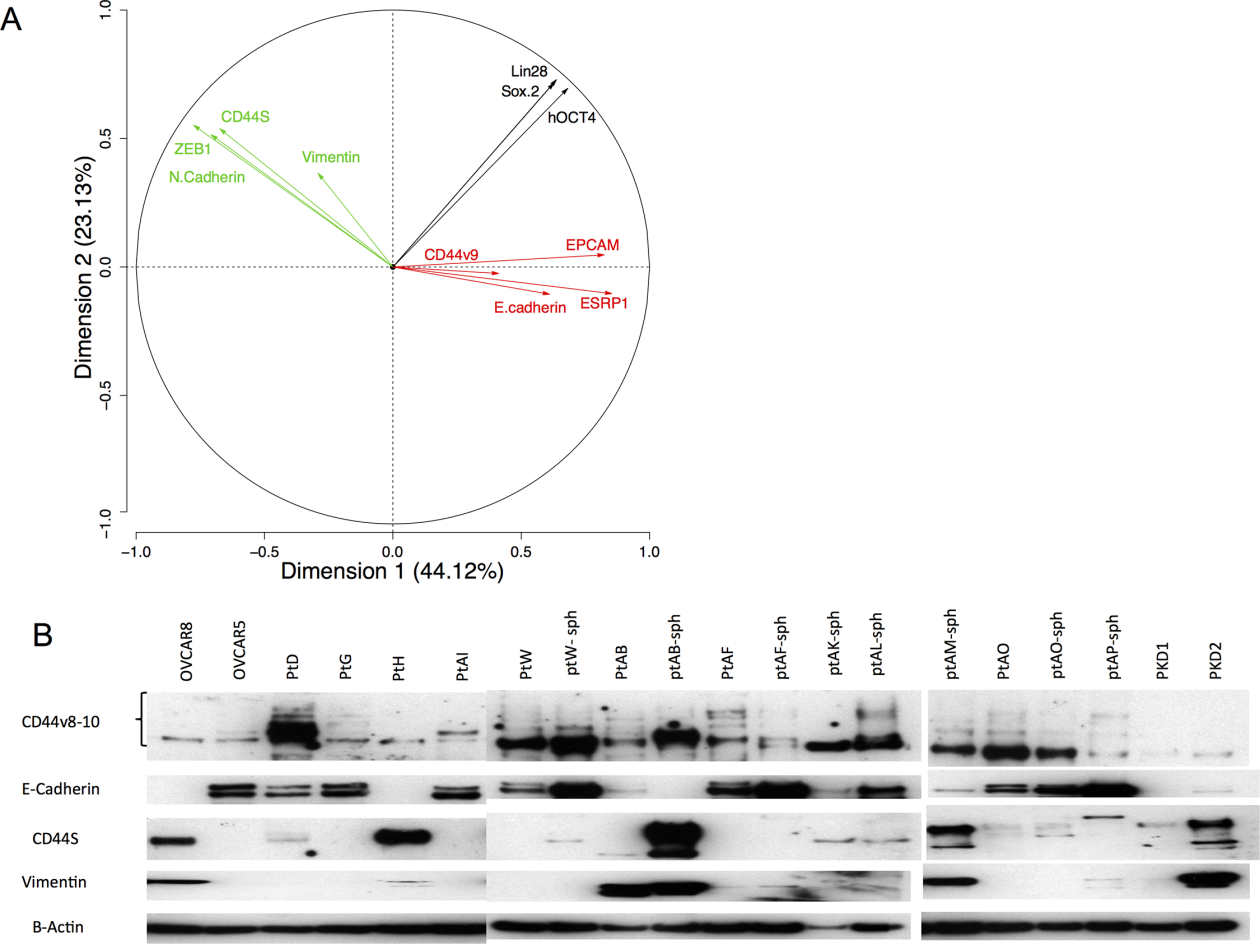
Expression of CD44v8-10 mRNA and protein, compared to epithelial markers and mesenchymal markers by Principal component Analysis (PCA) of qPCR and Western Blot. A) Principal component analysis using qPCR relative quantification of the expression of epithelial, mesenchymal, and pluripotency markers in primary ovarian cancer cell lines. Colored arrows indicate the directions for each group of markers. B) Western analysis of primary cell line protein lysates for expression of CD44v8-10 protein using a rat monoclonal antibody to human CD44v8-10.

We performed principle component analysis (PCA) (FactoMiner) on mRNA expression, as defined by qPCR values of 2^-ΔCt^ normalized to GAPDH, in a series of different epithelial (EPCAM, ESPR1, E-cadherin) and mesenchymal (ZEB1, Vimentin, N-cadherin) markers in a panel of 15 primary (ptAB, ptAB-sph, ptAF, ptAF-sph, ptW, ptW-sph, ptAO-sph, ptG, ptD, ptH, PKD1, PKD2, ptAL-sph, ptAP-sph, ptAM-sph) and 2 established ovarian cancer cell lines (OVCAR5, OVCAR8). The PCA confirmed that CD44v8-10 mRNA expression associates with other epithelial markers, whereas CD44s expression correlates with mesenchymal markers in ovarian cancer (Fig 4A). Expression of pluripotency markers (LIN28, SOX2, OCT4) was associated with neither epithelial nor mesenchymal markers, suggesting stemness is independent of epithelial or mesenchymal status (Fig 4A).

Similarly, expression of CD44v8-10 protein in patient-derived cell line lysates by western blot displayed a trend of co-expression of CD44v8-10 with E-cadherin, or CD44s with vimentin, in a mutually exclusive fashion (Fig 4B), confirming the qPCR data indicating that CD44v8-10 is an epithelial marker and CD44s a mesenchymal marker in high grade serous ovarian cancer.

**Fig 5:**
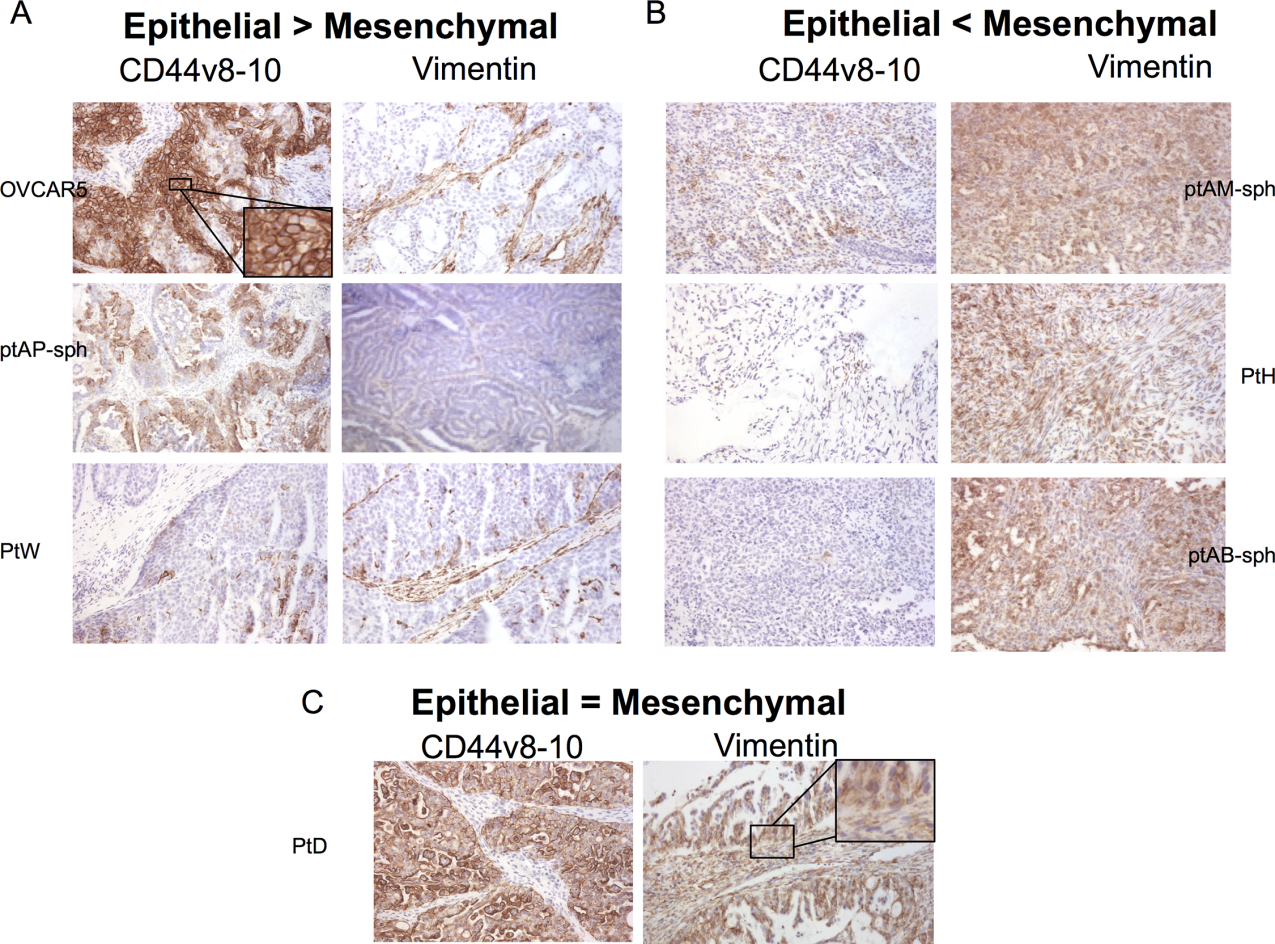
Pattern of expression of CD44v8-10 and EMT markers by immunohistochemistry in PDXa tumors. Antibody staining of tissue sections from tumors developed in vivo after injection of 1 to 5 million primary patient-derived ovarian cancer cells. Representative sections from tumors derived from OVCAR5, ptAP-sph, Pt W (A), ptAM-sph, ptH, ptAB-sph (B) and ptD (C) are shown. CD44v8-10, and Vimentin immunohistochemical stain is shown with hematoxylin counterstain at 100x magnification. CD44v8-10 labels the surface of epithelial cancer cells (insert), whereas vimentin labels stromal cells in more epithelial tumors and most cells in more mesenchymal tumors (insert). Tumors were grouped into epithelial (A), mesenchymal (B) or mixed phenotype (C) based on the staining.

To determine the spatial expression of CD44v8-10, and its relation to the epithelial or mesenchymal tumor histologies, patient-derived xenografts of ascites (PDXa’s) [16] were analyzed by immunohistochemistry (IHC). Overall, tumors grown *in vivo* recapitulated the epithelial or mesenchymal heterogeneity observed in the primary cell line *in vitro* (Fig 4). Tumors with a highly epithelial phenotype expressed low levels of vimentin, which was restricted to stromal cells (PtW, OVCAR5) and high levels of CD44v8-10 expression, which was specific to the cell surface of the cancer cells of the tumor (Fig 5A, insert). Conversely, the more mesenchymal tumors (PtH, PtAM spheres, and PtAB spheres) expressed vimentin in both the epithelial cancer cells as well as the stromal cells of the tumor, and had very low expression of CD44v8-10 (Fig 5B). Tumors of intermediate phenotype with both epithelial and mesenchymal characteristics had expression of CD44v8-10 and vimentin in the epithelial cells as well as the stroma (Fig 5C).

### Soluble CD44v8-10 is detectable in the supernatant of ascites samples and correlate with worse prognosis

**Fig 6:**
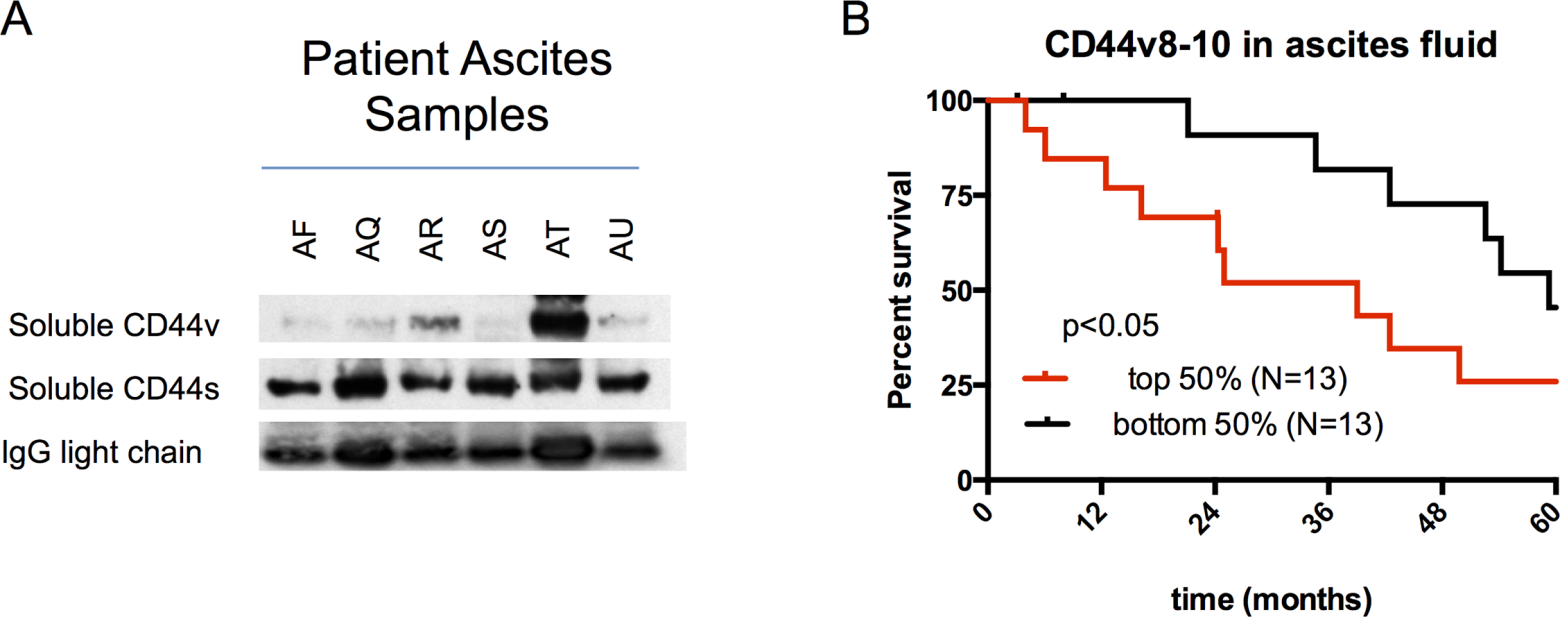
Detection of soluble cleaved extracellular domain of CD44v8-10 in patient ascites samples. A) Ascites samples (N = 28; 6 samples shown as representative) were obtained from patients undergoing therapeutic paracentesis, and probed by western blotting for CD44v8-10, CD44s, and anti human IgG light chain as a loading control. Protein densitometry was performed and samples were analyzed for levels of expression of soluble CD44v8-10 and separated into two equal groups of high and low CD44v8-10 levels. B) Kaplan-Meier survival curve of the patients stratified by CD44v8-10 abundance in the patient ascites shows high expression correlates with decreased survival (p = 0.0481). Bars represent censored data points.

We evaluated the potential to detect the soluble extracellular domain of CD44v8-10 as a cleaved fragment in ascites fluid supernatant using Western blot of 28 different patient ascites samples. Soluble cleaved CD44S is homogenously expressed in all ascites samples, including benign controls, suggesting it is a ubiquitous blood protein. Interestingly, the cleaved and shed soluble extracellular domain of CD44v8-10 was detectable in 27 out of 28 samples of patient ovarian ascites, with highly variable levels, but was not detectable in benign liver ascites, suggesting it may be cancer-specific. A representative western blot of 6 patient ascites is demonstrated in Fig 6A. The protein abundance of the soluble extracellular domain of CD44v8-10 was measured by densitometry of the Western blot bands and normalized for equal protein loading using IgG light chain density by Image J software analysis. After stratification of patients based on CD44v8-10 levels, we noted a significantly decreased survival in the patients with high levels of soluble CD44v8-10 (top 50%) with a median survival of 39 months versus 59.27 months for the bottom 50% (p = 0.0481) (Fig 6B).

## Discussion

Based on our analysis of The Cancer Genome Atlas (TCGA) RNAseq database of ovarian serous cystadenocarcinoma we hypothesized that expression of CD44 variants which include variable exons V8, V9, V10, the “epithelial” CD44 isoform, correlate with improved prognosis in ovarian serous cystadenocarcinoma. We confirmed this finding using an independent cohort of 210 patients with high-grade serous ovarian carcinoma in which we investigated cell surface expression of the transmembrane CD44v8-10 protein in a tumor tissue microarray. When patients were stratified based on expression levels to compare the top 10% and 20% to the bottom 10% and 20% respectively, we found a statistically significant improved survival in the highest expressing patients compared to the lowest expressing patients, with a 69% and 73% increase in median survival time respectively, which correspond to a difference of over 20 months. The predictive power of CD44v8-10 staining in the tumor is stronger in younger patients, suggesting it is best used for prognosis in patients under the age of 60. Increased survival was predicted by previous observations of a survival benefit associated with expression of CD44s and other full-length transmembrane CD44 variant isoforms[13, 14].

We hypothesized that the increased survival observed with high expression of cell surface transmembrane CD44v8-10 protein may be related to the more epithelial nature of those tumors. Using an unbiased approach based on principal component analysis of qPCR markers of mesenchymal and epithelial status, we found that CD44v8-10 correlates with epithelial markers such as E-cadherin and EpCAM, while CD44s correlates with mesenchymal markers such as vimentin in ovarian cancer. Previous studies using E-cadherin, EpCAM, and vimentin as tumor markers, have shown that epithelial differentiation of tumors is associated with improved prognosis in many cancer types including breast, ovarian, colorectal, prostate, and gastrointestinal [21], presumably due to the less invasive nature of these tumors. Expression of E-cadherin has been extensively studied since downregulation during epithelial to mesenchymal transition is associated with increased cancer cell migration and more metastatic tumors in ovarian cancer [22]. Conversely, EpCAM over-expression has been associated with both improved prognoses and favorable response to platinum based chemotherapy [23] further suggesting that epithelial differentiation leads to improved prognosis in ovarian cancer. Expression of the mesenchymal marker vimentin in epithelial cells of pancreatic and non-small cell lung carcinomas is correlated with poor prognosis [24, 25]. Similarly, in ovarian cancer, the presence of a higher percentage of vimentin positive cells in metastatic pleural effusions is associated with increased chemoresistance and poor initial response to chemotherapy [26]. These results suggest that the epithelial phenotype of ovarian cancer cells may be a favorable prognosticator in ovarian cancer, for which transmembrane CD44v8-10 may be a useful marker.

Interestingly, there is both intratumor and intertumor heterogeneity in the expression of epithelial and mesenchymal markers in our datasets suggesting the epithelial and mesenchymal phenotypes are not dichotomous but rather a continuum or an EM spectrum [27]. The data we obtained from primary tumor xenografts and from the primary cell lines from which the tumors were grown, suggest that some tumors can express epithelial markers such as CD44v8-10 protein alongside mesenchymal markers such as vimentin. Our observation supports the hypothesis that ovarian cancers are highly heterogeneous and can have intermediate characteristics in between the well-differentiated epithelial or mesenchymal phenotypes which may be important for prognosis, and for which additional markers of EM may be particularly useful [28, 29].

**Fig 7:**
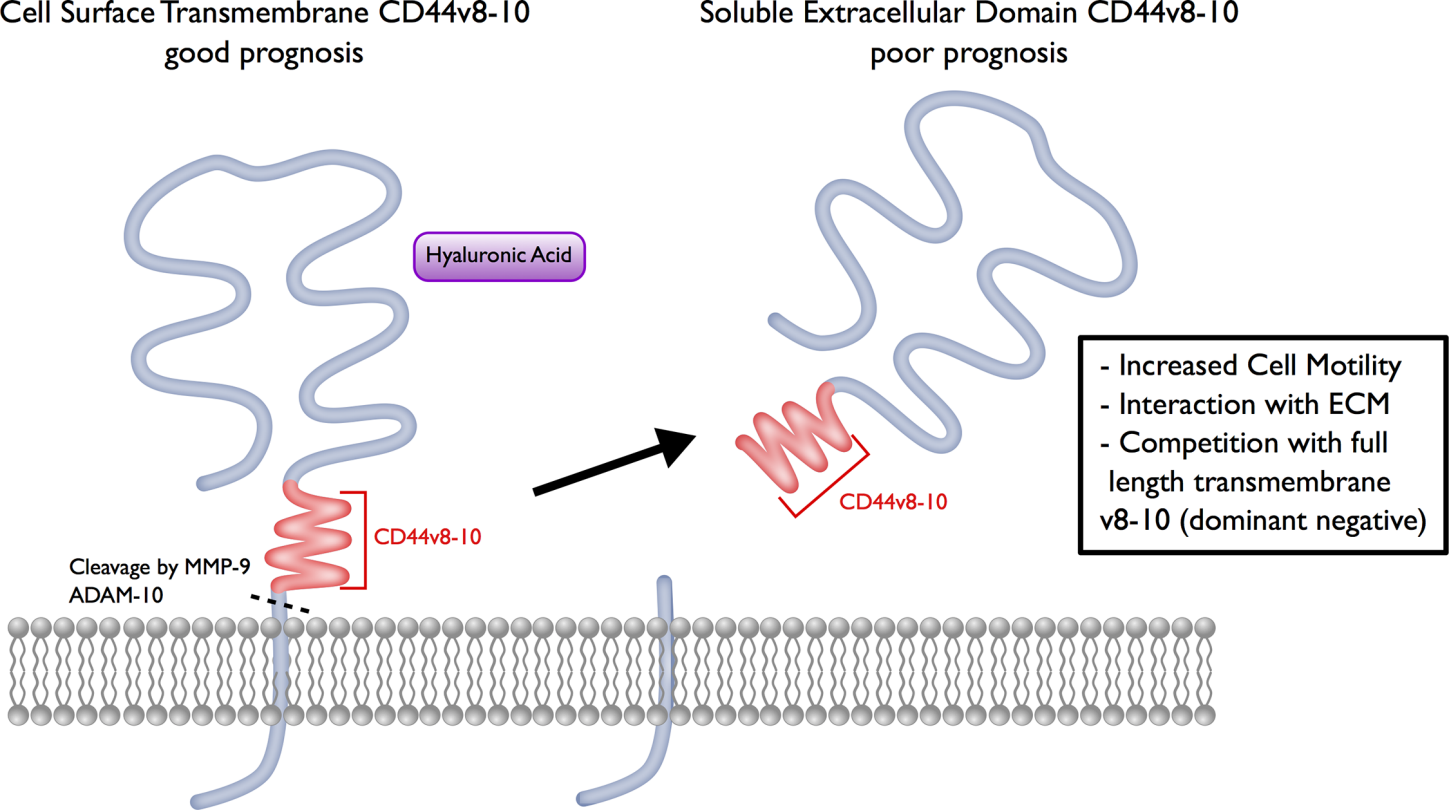
Surface expression of transmembrane CD44v8-10 protein or soluble cleaved CD44v8-10 extracellular fragments have distinct prognostic significances in ovarian cancer. High expression of the cell surface transmembrane CD44 containing variant exons v8-10 is associated with improved survival, which may be indicative of a more epithelial tumor. Presence of the cleaved soluble extracellular domain of CD44v8-10 in the ascites fluid is associated with poor survival, which may be indicative of a more metastatic tumor with high expression of proteases (MMP-9, ADAM-10). Higher levels of soluble CD44v8-10 may act as a dominant negative, preventing cells from reestablishing binding with the ECM, contributing to a more mesenchymal and metastatic phenotype.

It is important to differentiate between the proteolytically cleaved, soluble fragments and the intact protein of CD44v8-10 found on the cell surface. The presence of CD44v8-10 on the surface of the cancer cells and its association with epithelial differentiation, may confer a survival benefit in high grade serous ovarian cancer (Fig 7). However, we hypothesize that loss of CD44v8-10 from the cell surface could be implicated in epithelial to mesenchymal transition and more aggressive behavior of a tumor. Decreased surface expression of CD44v8-10 could be achieved by either downregulation of expression at the transcriptional level, an isoform switch regulated by splice factors away from variant exons v8-10, or as a result of increased proteolytic cleavage. For the latter, we have previously shown that CD44 cleavage is associated with tumor invasion and is mediated by the ADAM10 and ADAM17 proteases [30–32]. Furthermore the cleaved extracellular domain of CD44v8-10 may act as a dominant negative by preventing extracellular matrix binding (Fig 7). Previous attempts to detect CD44s and CD44 splice variants (V5, V6 and V7-8) in the serum of ovarian cancer patients did not reveal a significant prognostic effect of these molecules as a predictor of tumor burden or recurrence [33]; however, cleaved and soluble V8-10 isoforms have not been investigated in the serum or ascites of ovarian cancer patients. We were able to detect cleaved soluble CD44v8-10 in ascites of patients by western blot, and found an association with worse prognosis, suggesting this specific isoform may be a useful biomarker. We hypothesize that high cleavage rates of CD44v8-10 isoforms may be associated with more metastatic cancer cells which overexpress proteases, and since CD44v8-10 is highly specific to malignant ascites while CD44s is ubiquitous in patient fluids, soluble CD44v8-10 may represent a uniquely sensitive biomarker (Fig 7). Further studies should determine if the cleaved extracellular domain of CD44v8-10 is detectable in the serum of ovarian cancers patients by ELISA where it could be employed as a biomarker for prognosis, diagnosis, or tumor burden monitoring along with CA-125.

## Supporting Information

S1 Fig Correlation of all exons of CD44 in TCGA RNAseq data (RPKM). The bottom left quadrant shows the scatter plot and the top right the pearson correlation for each exon of CD44 including variable exons over all samples of the ovarian cancer RNAseq cohort. Exons 1–5 and 15–18 (the stable exons) strongly correlate to each other, as do exons 12–14 (variable exons 8–10). The other facultative exons 6–11 have lower degrees of correlation. This mirrors the relative abundance of CD44 isoforms in ovarian cancer in which the two most observed isoforms of CD44 are CD44s and CD44v8-10.
(TIFF)

S2 Fig Transcript abundance of various CD44 isoforms containing exons v8-v10 and their influence on survival in the TCGA ovarian cancer dataset. A) The density of expression of each CD44 isoform containing exons v8-10 is shown with the number of patients on the y axis and the relative abundance of the transcript in RPKM on the x axis. B) The Kaplan-Meier plots compare the top and bottom 10% of expressers in the patient population for each isoform. Isoforms are described in Fig 1A and S1 Table. Bars represent censored data points.
(TIFF)

S3 Fig Immunohistochemical and survival analysis of CD44v8-10 expression in Grade 3 Serous Ovarian Cancer Tumor Tissue Microarrays (TMAs). Overall Survival of patients with grade 3 serous ovarian carcinoma by CD44 variant expression (grade 2 excluded). Left- Highest and Lowest 10% expression variant and Right- Highest and Lowest 20% of expression of variant, demonstrates significant overall survival in the highest expressers with p = 0.038 and p = 0.014, respectively.
(TIFF)

S1 Table CD44 isoform transcript identifiers from the TCGA RNA-seqV2 data. (XLSX)

S2 Table High Grade Serous Ovarian Cancer Subgroup Characteristics sorted by Positivity Intensity (PI) score. (DOCX)
