# Supplementary figures and images for: CD44 Splice Variant v8-10 as a Marker of Serous Ovarian Cancer Prognosis
Source: PLoS One. 2016 Jun 2;11(6):e0156595. doi: 10.1371/journal.pone.0156595 (PMC4890777; doi:10.1371/journal.pone.0156595)

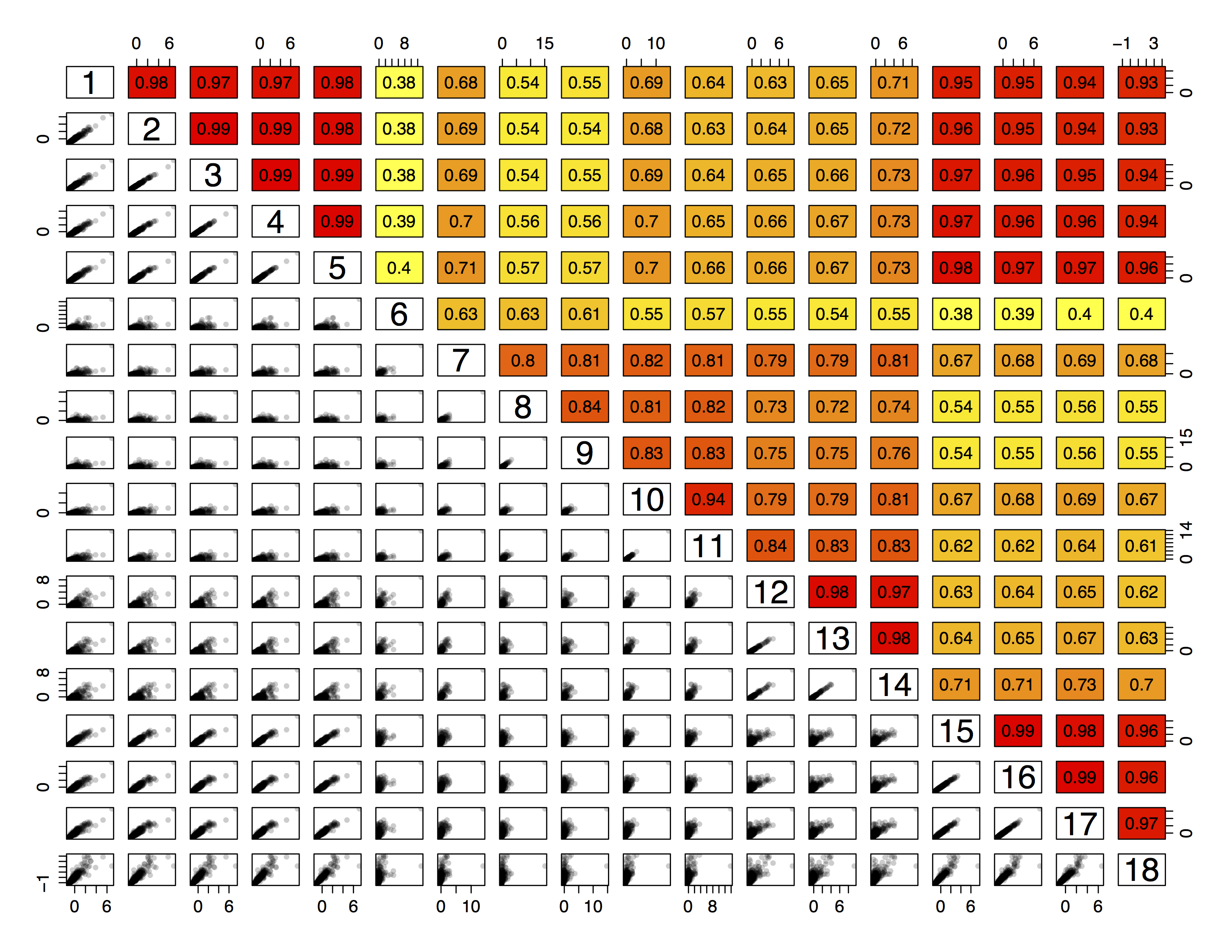

Supplement: S1 Fig — The bottom left quadrant shows the scatter plot and the top right the pearson correlation for each exon of CD44 including variable exons over all samples of the ovarian cancer RNAseq cohort. Exons 1–5 and 15–18 (the stable exons) strongly correlate to each other, as do exons 12–14 (variable exons 8–10). The other facultative exons 6–11 have lower degrees of correlation. This mirrors the relative abundance of CD44 isoforms in ovarian cancer in which the two most observed isoforms of CD44 are CD44s and CD44v8-10. (TIFF) [file pone.0156595.s001.tiff]

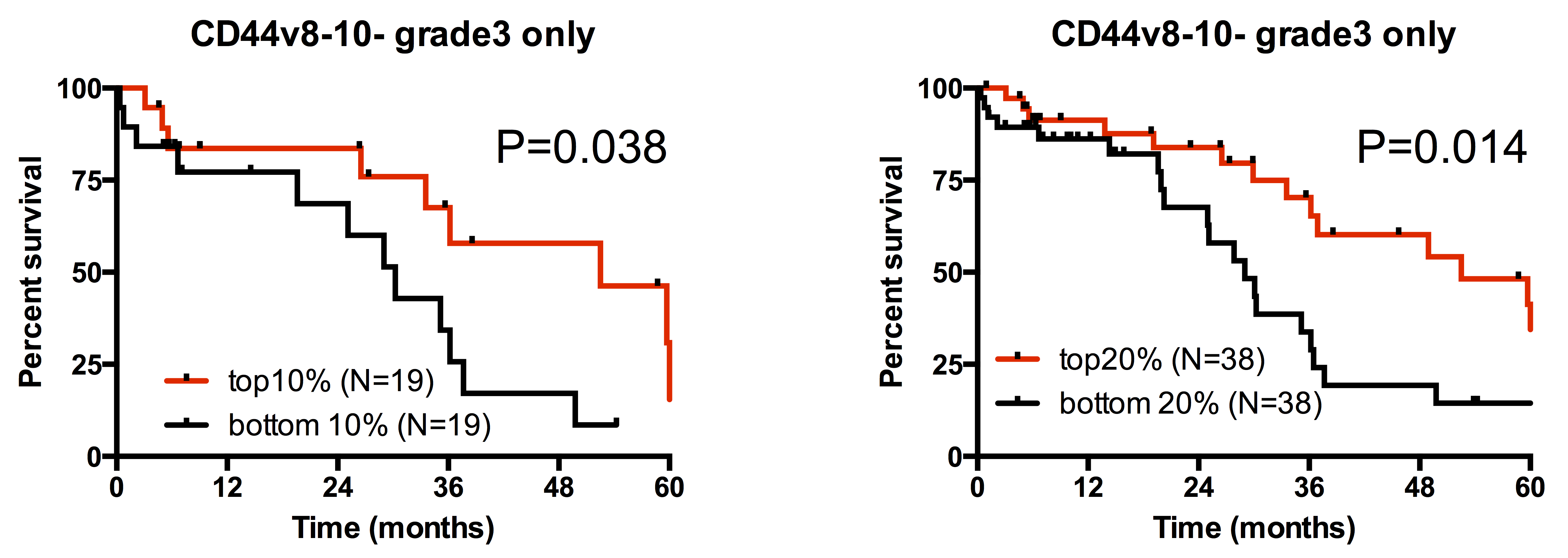

Supplement: S3 Fig — Overall Survival of patients with grade 3 serous ovarian carcinoma by CD44 variant expression (grade 2 excluded). Left- Highest and Lowest 10% expression variant and Right- Highest and Lowest 20% of expression of variant, demonstrates significant overall survival in the highest expressers with p = 0.038 and p = 0.014, respectively. (TIFF) [file pone.0156595.s003.tiff]
