# Supplementary material for: CD44 Splice Variant v8-10 as a Marker of Serous Ovarian Cancer Prognosis
Source: PLoS One. 2016 Jun 2;11(6):e0156595. doi: 10.1371/journal.pone.0156595 (PMC4890777; doi:10.1371/journal.pone.0156595)
Supplement: S2 Table — (DOCX) [file pone.0156595.s005.docx]

|  | **Top 10%**  **(n=21)** | **Bottom 10%**  **(n=21)** | **Top 20%**  **(n=42)** | **Bottom 20%**  **(n=42)** |
| --- | --- | --- | --- | --- |
| **Age** |  |  |  |  |
| <50  >50 | 4  17 | 2  19 | 9  33 | 5  37 |
| **FIGO** |  |  |  |  |
| Unknown  Stage III  Stage IV | 1  19  1 | 4  10  7 | 3  35  4 | 5  28  9 |
| **Grade** |  |  |  |  |
| 2  3 | 5  16 | 0*  21 | 8  34 | 1*  41 |
| **Debulking Status** |  |  |  |  |
| Unknown  Suboptimal  optimal | 3  5  13 | 3  5  13 | 5  11  26 | 4  10  28 |
